# SEOM clinical guidelines to primary prevention of cancer (2018)

**DOI:** 10.1007/s12094-018-02016-4

**Published:** 2019-01-03

**Authors:** J. Bayo, R. Molina, J. Pérez, E. Pérez-Ruíz, J. Aparicio, C. Beato, J. P. Berros, M. Bolaños, B. Graña, A. Santaballa

**Affiliations:** 1 Servicio de Oncología Médica, Hospital Juan Ramón Jiménez de Huelva, Ronda Exterior Norte, s/n, 21005 Huelva, Spain; 2 Servicio de Oncología Médica, Hospital, Universitario Príncipe de Asturias, Madrid, Spain; 3 Servicio de Oncología Médica, Hospital Virgen de los Lirios, Alcoy, Spain; 4 Servicio de Oncología Médica, Complejo Hospital Costa del Sol, Marbella, Spain; 5 Servicio de Oncología Médica, Hospital Universitari i Politècnic la Fe (Valencia), Valencia, Spain; 6 Servicio de Oncología Médica, Complejo Hospitalario Regional Virgen Macarena, Seville, Spain; 7 Servicio de Oncología Médica, Hospital, Universitario Central de Asturias, Asturias, Spain; 8 Servicio de Oncología Médica, Complexo Hospitalario Universitario A Coruña (CHUAC), Coruna, Spain

**Keywords:** Primary prevention, Cancer, Healthy lifestyle

## Abstract

Cancer is the leading social and healthcare problem of the twenty-first century. The aim of primary prevention is to decrease the incidence of cancer by avoiding the known causes and risk factors. Nevertheless, it has been estimated that cancer diagnoses could be halved through primary prevention measures. A comprehensive review of the scientific evidence regarding the main carcinogens and risk factors and primary prevention recommendations have been put forth based on this evidence. The GRADE scale has been used to classify the grade of evidence. We present the scientific evidence and recommendations for primary prevention of the major modifiable risk factors: smoking, alcohol, diet, obesity, physical activity, occupational and environmental factors, ultraviolet radiation, infections, and socioeconomic factors. Primary prevention is a simple, effective means to lower the incidence of cancer. Preventive measures must be circulated in the fight against cancer.

## Introduction

Cancer is one among the top causes of morbi-mortality around the world. Populational data point toward its incidence increases in the coming decades, up to some 24 million cases in 2035. In Spain, it has been estimated that there will be 315,413 cases by the year 2035. Furthermore, cancer is also the second leading case of death in our country, with 112,939 deaths in 2016–27.5% of all deaths for that year.

While some authors advocate that many neoplasms would be due to random mutations during a cell division, the vast majority of the literature maintains that a significant proportion of these cases could be avoided by changing lifestyle-related factors [1]. Primary prevention seeks to avert the appearance of the disease by controlling these factors through strategies aimed at decreasing exposure to hazardous factors or encouraging exposure to protective factors [2].

In these guidelines, we will review the main, modifiable, risk factors, susceptible to action from the perspective of primary prevention.

## Smoking

Cigarette smoking is the number one preventable cause of cancer. Tobacco has been estimated to cause more than 1 million cancer deaths in the world. The carcinogenicity of cigarette smoke and smokeless cigarettes has been widely proven (grade IA). In laboratory animals, 62 carcinogenic agents have been reported, 15 of which are conclusively carcinogenic in humans. Any number of international, prospective, epidemiological, and case-control studies carried out with a million subjects has confirmed these findings [3].

At least 14 types of cancers have been reported to be caused by tobacco use—the most common and lethal of them being lung cancer. Its main determinant is the time during which the person smoked, but the risk also increases with the number of cigarettes smoked. Smoking multiplies the risk of all histological types of lung cancer in both sexes. Quitting smoking at any age prevents the growing risk of developing the disease associated with active smoking, although the risk continues to be greater for years after quitting compared to individuals who have never smoked.

Tobacco smoke is also a leading cause of transitional cell carcinomas of the bladder, ureter, and renal pelvis. There is a causal association between cancer of the mouth, larynx, paranasal sinuses, pharynx, and esophagus, the risk of which increases significantly with the simultaneous consumption of alcohol. Other smoking-related neoplasms include cancers of the pancreas, stomach, liver, uterine cervix, and myeloid leukemia. Passive smokers are at a greater risk for developing lung, laryngeal, and pharyngeal cancers.

## Consumption of alcohol

Together with body weight, the consumption of alcohol is the number one dietary risk factor for cancer. Acetaldehyde, an active metabolite of ethanol, is the main carcinogen in alcoholic beverages [4]. The biological, animal, and epidemiological evidence has caused alcohol to be included in group 1 of the classification of carcinogen in humans (grade IA). There is sufficient proof to conclude that the consumption of alcohol plays a causal role in the development of squamous cell carcinomas of the mouth, pharynx, larynx, and esophagus (these tumors present the greatest evidence available and the effect is synergistic with tobacco), and in liver, colon, rectal, and female breast cancers. There is also evidence that points to a probable relationship with cancers of the stomach and pancreas.

The mechanism includes direct damage to the cells of the upper gastrointestinal tract, modulation of DNA methylation (that increases susceptibility to mutations), and the increase in acetaldehyde (that stimulates proliferation of epithelial cells and forms DNA adducts). The relationship between alcohol and cancer risk has been proven to be modified by the genetic variations in carbon and acetaldehyde metabolic pathways. The risk of developing cancer increases linearly with growing levels of average daily consumption. The magnitude of this risk varies according to the type of cancer, and no minimum threshold of consumption has been detected for the risk of cancer. The association with breast cancer is striking, insofar as a single drink per day is associated with a small, albeit significantly increased risk. It appears that adequate intake of folates can exhibit a protective effect. Diets poor in fruits and vegetables enhance the carcinogenic effect in otorrhinolaryngeal squamous cell carcinomas. For most tumor sites, no differences have been found based on the type of alcoholic beverage consumed.

## Diet

It is estimated that 30–35% of all malignant tumors are diet related and there are enough scientific data to endorse dietary recommendations in cancer prevention [5]. The relationship between diet and cancer is common knowledge, albeit with a significant influence of biased data and commercial interests. Table 1 displays dietary recommendations for the prevention of cancer put forth by three European and North American organizations; they are based on robust evidence and are mutually consistent.

**Table 1:** Dietary recommendations adapted from the World Cancer Research Fund (WCRF), the American Cancer Society (ACS), and the European Code Against Cancer

World cancer research fund (WCRF)	American cancer society (ACS) Kushi et al. (2012)	European code against cancer Norat et al. (2015)
Maintain a healthy weight throughout life.	Stay as lean as possible throughout life without being underweight; avoid excess weight gain at all ages; if you are overweight or obese, lose weight	Maintain a healthy weight throughout life
Limit calorie-laden foods and avoid sugar-sweetened beverages	Choose foods and drinks in amounts that help you get to and maintain a healthy weight	Limit high-calorie foods.
Base your daily diet on vegetables, with whole grains, legumes, non-starchy vegetables, and fruit at every meal	Choose whole grains instead of refined grain products	Eat plenty of whole grains, legumes, vegetables, and fruits
Limit the amount of salt and salty foods	Eat at least two and a half cups of vegetables and fruits everyda	Avoid sugar-sweetened beverages
Limit red meat and avoid processed meats	Limit the amount of processed and red meats	Limit red meat and avoid processed meats
Limit your intake of alcoholic beverages	If you drink alcohol, limit it to one drink per day for women or two per day for men	Limit or avoid the alcoholic beverages
Do not use dietary supplements as cancer prevention	Dietary supplements do not lower the risk of cancer; if you take vitamins or minerals, do not exceed 100% of the daily recommended amount for each component	

(Our own source)

Most of the data regarding nutrition come from retrospective meta-analyses of case-control studies and prospective cohort studies. Prospective, randomized studies are more complex to perform, basically given the difficulty inherent in guaranteeing and verifying patients’ long-term compliance with a specific nutritional diet [6].

In a recent systematic review and meta-analysis of a total of 93 studies that included more than 85,000 cases, 100,000 controls, and 200,000 individuals exposed, the greatest evidence supported an association between the models of healthy diet and the reduction of colon and breast cancer risk (especially with negative hormone receptors in postmenopausal women), and the association between a pattern of an unhealthy diet and increased risk of colon cancer (grade 1B) [7]. There is less evidence, and what evidence does exist is based on the case-control studies that point to a pattern of unhealthy diet and the risk of cancer of the upper airway digestive tract, pancreas, ovary, endometrium, and prostate. The results suggest a potential role of diet in certain types of cancer, but the evidence is less conclusive and might be mediated by other lifestyle factors.

Another systematic revision and meta-analysis (PRISMA) [8] evaluated the association between adherence to established dietary and physical activity guidelines and the general incidence and mortality due to cancer. A total of 2033 potentially relevant studies were reviewed and 12 prospective publications that followed the recommendations of the American Cancer Society/World Cancer Research Foundation (ACS/WCRF) were analyzed. They found consistently lower risks for certain specific tumor sites (grade 1B). High versus low compliance with the recommendations was significantly associated with a decrease between 10 and 61% in the overall incidence of colorectal cancer in both men and women. They also detected consistent, significant decreases in the incidence of breast cancer (19–60%) and endometrial cancer (23–60%). The results for lung cancer were ambiguous. No significant relationship was detected for cancer of the ovary or prostate.

The International Agency for Research on Cancer (IARC) has evaluated more than 800 epidemiological studies that analyzed the association between cancer and the consumption of red or processed meats in several countries. Most of the epidemiological data referred to colorectal cancer, and reported a positive correlation between this tumor and the consumption of red meat and processed meat in 14 and 12 cohort studies, respectively. Likewise, there were data for more than another 15 types of cancers [9]. A positive association was found in cohort studies and populational case-control studies between the consumption of red meat and cancer of the pancreas and prostate and between the consumption of processed meat and gastric cancer. The working group classified the consumption of processed meat as carcinogenic for humans (group 1), based on there being enough evidence for colorectal cancer (grade 1A). In addition, a positive association was found between the consumption of processed meat and cancer of the stomach. The consumption of red meat was qualified as probably carcinogenic in humans (group 2) (grade 1B).

## Obesity and cancer

More than 1000 epidemiological studies and meta-analyses have been published that observe an increased risk of different kinds of cancer associated with overweight and obesity. The general conclusion was that not being overweight or obese lowers the risk of developing several tumor types, suggesting a preventive effect against cancer as a result of an attempt to lose weight, although the evidence in humans must be confirmed by subsequent studies.

After the comprehensive analysis of the evidence, the IARC has concluded that the absence of overweight and/or obesity decreases the likelihood of developing cancer of the colon, esophagus (adenocarcinoma), renal cell carcinoma, breast and uterine cancer in postmenopausal women, cancer of the cardias, liver, gall bladder, pancreas, ovary, thyroid, as well as multiple myeloma and meningioma [10]. The association between a high BMI and lower overall survival in breast cancer has likewise been proven, although the evidence is inconsistent with respect to other tumors.

Weight loss following bariatric surgery lowers the risk of breast and endometrial cancers, according to the data from observational studies, although number and quality of those studies are insufficient to make a robust evaluation.

## Physical activity

The WHO defines physical activity (PA) as any bodily movement produced by skeletal muscles that requires energy expenditure [11]. Lack of PA is a significant factor in mortality and is directly responsible for 6% of all the deaths worldwide [12]. The evidence that maintains the impact of PA in lowering cancer risk comes, in large part, from observational studies (grade IC). Table 2 presents the evidence available in the best-studied tumor subtypes.

**Table 2:** Evidence available regarding the benefit of physical activity in colorectal, breast, and endometrial cancers

Colorectal cancer	Meta-analysis of 52 epidemiological studies that examined the association between PA and the risk of colon cancer found a 24% lower risk of colon cancer in those individuals who were more physically active
	Pooled analysis of data from 12 prospective cohort studies on PA performed during free time achieved a 16% decrease in the risk of colon cancer in the group of individuals who were more active and in proximally located tumors
	PA is also associated with a lower risk of adenomas
Breast cancer	Meta-analysis of 31 prospective studies revealed an average 12% risk reduction of breast cancer associated with PA
	Regular PA has been associated with a decrease in breast cancer in both premenopausal as well as postmenopausal women, although the decrease is greater in postmenopausal women
	Women who increase PA after menopause can also lower their risk of breast cancer than those who do not
Endometrial cancer	A meta-analysis of 33 studies yielded an average 20% decrease in the risk of endometrial cancer associated with high-intensity PA, as opposed to low-intensity PA
	The association between PA and the risk of endometrial cancer might reflect the effect of PA on obesity, a known risk factor for endometrial cancer, although the level of evidence is low

(Own source)

## Occupational and environmental factors

The occupational carcinogenic risk due to exposure to chemical, physical, or biological agents has been widely documented. There are currently 25 chemical agents or groups or mixtures thereof in the area of occupational exposure that have been established as human carcinogens and another 25 as probable carcinogens.

Some of these agents (asbestos, silica, and heavy metals) are currently found in many jobs, but others have only historical value (mustard gas; 2-naphthylamine). Their carcinogenicity has been proven in animal experiments, whereas, in humans, the evidence is less conclusive and based on epidemiological studies (grade 2C).

An increased risk of cancer can often be associated with a group of agents or professional occupation (Table 3). Between 4 and 5% of the cancer in developed countries has been estimated to be attributable to occupational exposures, with lung cancer heading the list [13, 14].

**Table 3:** Chemicals classified as human carcinogens (IARC group 1) for which exposures are mostly occupational. (Source: Fuente IARC)

Agent	Cancer site/cancer	Main industry/use
4-Aminobiphenyl	Bladder	Rubber manufacture
Arsenic and arsenic compounds*	Lung, skin	Glass, metals, pesticide
Asbestos	Lung, pleura, peritoneum	Insulation, filter material, textiles
Benzene	Leukemia	Solvent, fuel
Benzidine	Bladder	Dye/pigment manufacture
Beryllium and beryllium compounds	Lung	Aerospace industry/metals
Bis(chloromethyl) ether	Lung	Chemical intermediate/by-product
Cadmium and cadmium compounds	Lung	Dye/pigment manufacture
Chloromethyl methyl ether	Lung	Chemical intermediate/by-product
Chromium[VI] compounds	Nasal cavity, lung	Metal plating, dye/pigment manufacture
Coal tar pitches	Skin, lung, bladder	Building material, electrodes
Coal tars	Skin, lung	Fuel
Ethylene oxide	Leukemia	Chemical intermediate, sterilizing agent
Mineral oils, untreated and mildly treated	Skin	Lubricants
Mustard gas (sulfur mustard)	Pharynx, lung	War gas
2-Naphthylamine	Bladder	Dye/pigment manufacture
Nickel compounds	Nasal cavity, lung	Metallurgy, alloys, catalyst
Shale oils	Skin	Lubricants, fuels
Silica, crystalline	Lung	Stone cutting, mining, foundries
Soots	Skin, lung	Pigments
Strong inorganic acid mists containing sulfur	Larynx, lung	Metal, batteries
Talc containing asbestiform fibers	Lung	Paper, paints
2,3,7,8-Tetrachlorodibenzo-para-dioxin (TCDD)	Several organs	Contaminant
Vinyl chloride	Liver	Plastic monomer
Wood dust	Nasal cavity	Wood industry

*This evaluation applies to the group of chemicals as a whole and not necessarily to all individual chemicals within the group

Environmental pollution refers specifically to cancer-causing environmental factors such as air, water, and soil pollutants. Human beings lack control over their level of exposure to these pollutants, and it is estimated that between 1 and 4% of cancer cases are attributable to these pollutants in the developed countries (grade 2C). The environmental carcinogens with the most evidence available are asbestos (non-occupational exposure), toxins in urban air, in the air inside homes, and in drinking water due to chlorination products [13, 14].

## Ultraviolet radiation

Ultraviolet radiation (UVR) has been classified by the IARC as a group 1 carcinogen. UVR can come from natural sources, such as the sun, or from artificial sources such as tanning beds, and is the number one carcinogen for developing skin cancer (SC) [15], the incidence of which is rising at an alarming rate. The different patterns of exposure to the sun are associated with different types of SC. Chronic or occupational exposure is associated with squamous cell carcinomas and some types of malignant melanoma (MM), whereas acute intermittent exposure and sunburns during childhood and adolescence are associated with basal cell carcinomas and MM [17]. Although there are other risk factors for SC, such as each person’s photo-type, family history, or solid-organ transplant, UVR is the main modifiable factor to lower the incidence of this type of tumor. Table 4 shows a summary of the preventive actions and grade of recommendation for each [16, 17].

**Table 4:** Ultraviolet radiation, preventive actions, and grade of recommendation

Preventive action	Grade of recommendation
*Reduce sun exposure in children and adolescents with light photo-type skin and who sunburn easily	1A
*Reduce sun exposure in adults over the age of 24 years	1B
Avoid the use of tanning lights or beds	1A
**Stay in the shade during the mid-day hours (10 am–4 pm)	1B
Use sunscreen (SPF > 15)	1A
Wear clothing, hat, and glasses	1C

(Own source)

*SPF* sun protection factor

*Elimination of exposure to sun light is not recommended, since, as we know, it is needed to synthesize vitamin D; moreover, it would decrease exercise that entails clear health benefits and the prevention of other diseases

**If you must be exposed to sun ultraviolet radiation, seeking natural (trees) or artificial shade is the best alternative to decrease exposure to ultraviolet radiation

## Infections

There is scientific evidence as to the role of infections due to different microorganisms in tumor development and proliferation. Infections cause one out of every five cancers through mechanisms of immunosuppression, cell transformation, and disturbances of the cell cycle [18].

The most widely reported associations between infections and tumor disease with the highest level of evidence are the following:

- human papilloma virus (HPV) with cancer of the uterine cervix, anogenital cancer, and squamous cell carcinoma of the head and neck [19];
- hepatitis B virus (HBV) and hepatitis C virus (HCV) with hepatocellular carcinoma [20];
- HTLV-1 virus with T cell leukemia in adults;
- HIV-1 virus with Kaposi sarcoma, non-Hodgkin lymphoma;
- herpes 8 virus with Kaposi sarcoma and lymphomas;
- Epstein-Barr virus (EBV) with Burkitt’s lymphoma;
- helicobacter pylori bacteria with gastrointestinal neoplasms, especially MALT lymphomas and gastric carcinoma;
- different trematoda with cholangiocarcinoma and hepatocellular carcinoma.

These infections are spread through blood and other bodily fluids, which open the door for prevention (screening of these infections in the blood, prevention of shared use of syringes in drug users, etc.). HBV and HPV vaccinations have proven to be efficacious. Vaccination against the human papilloma virus is recommended in boys and girls, and in male and female young people who have not been vaccinated.

Antiretroviral therapy against HIV has lowered the incidence of HIV-related lymphomas. Antiretroviral treatment in patients with HBV has decreased the incidence of hepatocarcinoma in these patients.

## Socioeconomic factors

In 2005, a review was carried out of the studies that address the influence of socioeconomic determinants on the incidence and mortality of cancer in North America. Despite the magnitude of the problem, it was concluded that these studies in this regard are lacking. One of the key points is the lack of homogeneity in how the expression “socioeconomic inequalities” is defined. In this review, it was proposed that it be taken to mean the result of inequalities associated with adverse living and/or working conditions, inadequate or insufficient health care, as well as with experiences and policies related to socioeconomic position and discrimination (grade 2B) [21].

Overall, the incidence of cancer is higher in the less developed countries, albeit there are also differences within the remaining countries with a significant increased risk of malignant tumors such as those affecting the lung, stomach, upper gastrointestinal tract, and cervix among socially underprivileged people (grade 2B) [22].

In Spain, a systematic review of the literature was conducted in 2010 that included studies performed on this subject in our country, where there is also a paucity of these studies. Generally speaking, there is an excessive risk for the incidence of all cancers among the more underprivileged social strata (grade 2B) [23].

The WHO’s Commission for Social Determinants of Health identifies different actions, such as improving daily living conditions, tackling the unequal distribution of power, money, and resources, quantifying the problem, and evaluating the results of the social and healthcare actions undertaken. In Europe, strategies and policies are being implemented in an attempt to counteract social inequalities with a growing awareness of the problem of public health [24].

## Conclusion

Primary prevention is the easiest and most effective way to prevent cancer. Table 5 provides a summary of the main risk factors and healthy lifestyle recommendations in primary prevention of cancer. The current version of the European Code against Cancer brings together the leading primary prevention measures in a text targeting the general population [25].

**Table 5:** SEOM guideline recommendations for primary prevention of cancer

Factor	Types of cancers	Grade	Preventive measures
Tobacco	Lung, bladder, ureter, pelvis renal, cavity oral, larynx, paranasal sinuses, pharynx, esophagus, pancreas, stomach, liver, uterine cervix, myeloid leukemia…	IA	Prevent people from starting to smoke
			Quit smoking in smokers
			Protect passive smokers
Alcohol	Squamous cell carcinomas of the cavity oral, pharynx, larynx, and esophagus (synergy with tobacco)	IA	Limit consumption (the minimum threshold remains unknown)
	Less evidence: liver, colon, rectum, and female breast		Appropriate diet
	Probable: stomach and pancreas		
Diet	Colon, breast	IB	Limit calorie-dense meals and sugar-sweetened beverages.
	Limited evidence: cancer of the upper airway digestive tract, pancreas, ovary, endometrium, and prostate		
			Limit the intake of red meat and avoid processed meats
			Eat abundant whole grains, legumes, vegetables, and fruits
Obesity	Colon, esophagus (adenocarcinoma), renal, breast, endometrial	IB	Appropriate weight control
			Weight loss in obese individuals
	Less evidence: cardias, liver, gall bladder, pancreas, ovary, thyroid, as well as multiple myeloma and meningioma		
Physical activity	Colon, breast, endometrial	IB	Stimulate physical activity in the population
Occupational and environmental risk	Listed in Table 3	IA	Avoid carcinogens Occupational protection
Ultraviolet radiation	Skin cancer (basal cell and squamous cell carcinomas) and malignant melanoma	IA/IB	Reduce exposure to the sun, especially in children and adolescents,
			Use sunscreen
			Avoid the use of tanning lamps or beds
Infections	(HPV) with cancer of the uterine cervix, anogenital cancer, and squamous cell carcinoma of the head and neck	IA	HBV and HPV vaccination
			Diagnosis and treatment of infections
	Hepatitis B virus (HBV) and hepatitis C virus (VHC) with hepatocellular carcinoma [26]		
	HTLV-1 virus with T cell leukemia in adults		
	HIV-1 virus with Kaposi sarcoma, non-Hodgkin lymphoma		
	Herpes 8 virus with Kaposi sarcoma and lymphomas		
	Epstein–Barr Virus (EBV) with Burkitt’s lymphoma		
	Helicobacter pylori with MALT lymphomas and gastric carcinoma		
	Trematoda with cholangiocarcinoma and hepatocellular carcinoma		
Socioeconomic factors	Cancer of the lung, stomach, upper gastrointestinal tract, cervix…	IIA	Policies to fight social inequalities
